# Supplementary material for: Nonequilibrium charge-density-wave order beyond the thermal limit
Source: Nat Commun. 2021 May 3;12:2499. doi: 10.1038/s41467-021-22778-w (PMC8093280; doi:10.1038/s41467-021-22778-w)
Supplement: Supplementary file 1 — Supplementary Information [file 41467_2021_22778_MOESM1_ESM.pdf]

Supplementary Information for "Nonequilibrium Charge-Density-Wave Order Beyond the Thermal Limit"

J. Maklar[1,*], Y. W. Windsor[1], C. W. Nicholson[1,†], M. Puppin[1,‡], P. Walmsley[2,3], V. Esposito[3,4], M. Porer[4], J. Rittmann[4], D. Leuenberger[5], M. Kubli[6], M. Savoini[6], E. Abreu[6], S. L. Johnson[6], P. Beaud[4], G. Ingold[4], U. Staub[4], I. R. Fisher[2,3], R. Ernstorfer[1], M. Wolf[1], and L. Rettig[1,*]

[1] *Fritz-Haber-Institut der Max-Planck-Gesellschaft, Faradayweg 4-6, D-14195 Berlin, Germany*
[2] *Geballe Laboratory for Advanced Materials and Department of Applied Physics, Stanford University, CA 94305, USA*
[3] *Stanford Institute for Materials and Energy Sciences, SLAC National Accelerator Laboratory, 2575 Sand Hill Road, Menlo Park, CA 94025, USA*
[4] *Swiss Light Source, Paul Scherrer Institut, CH-5232 Villigen PSI, Switzerland*
[5] *Department of Physics, University of Zürich, CH-8057 Zürich, Switzerland*
[6] *Institute for Quantum Electronics, Physics Department, ETH Zürich, CH-8093 Zürich, Switzerland*
[*] *Correspondence should be addressed to J.M. (maklar@fhi-berlin.mpg.de) or L.R. (rettig@fhi-berlin.mpg.de)*
[†] *Current address: Department of Physics and Fribourg Center for Nanomaterials, University of Fribourg, Chemin du Musée 3, CH-1700 Fribourg, Switzerland*
[‡] *Current address: Laboratory of Ultrafast Spectroscopy, ISIC, Ecole Polytechnique Fédérale de Lausanne (EPFL), CH-1015 Lausanne, Switzerland*

March 17, 2021

Supplementary Figures

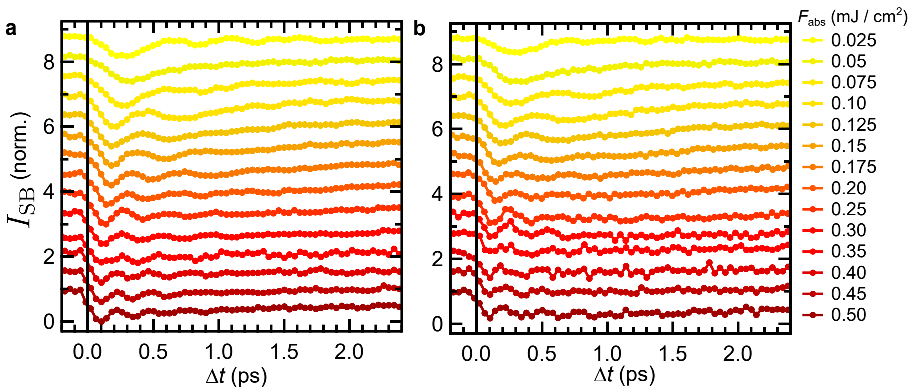

Supplementary Fig. 1: Shadow band intensity dynamics. Shadow band intensities extracted from (**a**) the nested (gapped) region of the FS, see box 2 in Fig. 2b, and (**b**) from the imperfectly nested (metallic) region, see box 3. Despite a slightly lower data quality in **b**, both shadow bands exhibit identical behaviour over the entire fluence range. The curves are vertically offset for clarity. For each curve, an intensity background extracted from a box slightly horizontally offset from the shadow band position is subtracted. Further, all curves are normalized by their respective intensities before excitation.

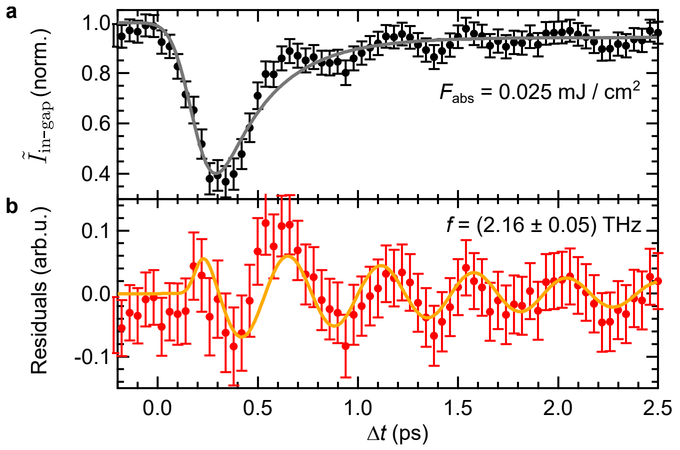

Supplementary Fig. 2: Electronic AM dynamics. (**a**) Time evolution of the inverted in-gap intensity after weak excitation. The grey line marks a double-exponential fit convolved with a Gaussian. (**b**) Fit residuals showing a pronounced amplitude mode in agreement with previous trARPES experiments[1,2], with a damped sinusoidal fit (orange curve). The error bars correspond to one standard deviation from electron counting statistics.

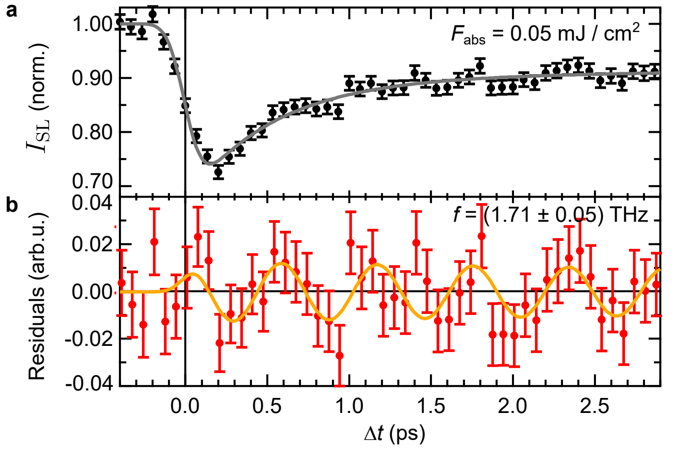

Supplementary Fig. 3: Structural AM dynamics. (**a**) Time evolution of the normalized (2 10 1+q_{CDW}) SL peak intensity after weak excitation. The grey line marks a double-exponential fit convolved with a Gaussian. The error bars correspond to one standard deviation from photon counting statistics. (**b**) The fit residuals indicate weak oscillations superimposed on the exponential decay, corresponding to a phonon mode that strongly couples to the CDW amplitude mode at 100 K, in agreement with previous optical and trXRD studies[3–6], with a damped sinusoidal fit (orange curve).

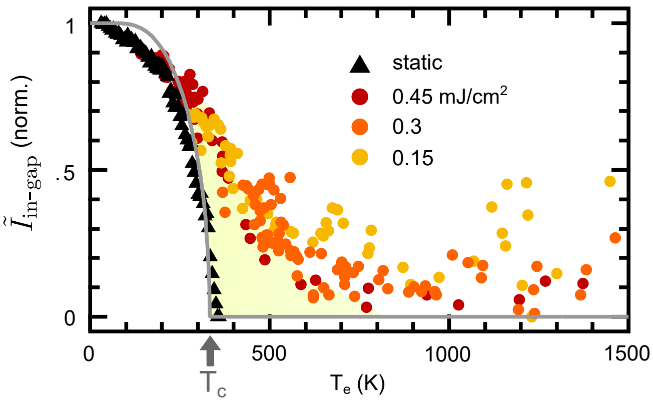

Supplementary Fig. 4: *T*-dependent CDW recovery dynamics. Inverted in-gap intensity of the dynamic CDW recovery versus extracted electronic temperatures for selected fluences. For reference, the static T-dependence is shown (black) with the BCS-like mean-field curve (grey). For clarity, the values of the dynamic traces for t <200 fs (initial CDW melting) are omitted. The dynamic curves follow a universal recovery behaviour over a wide range of fluences. The region of nonthermal CDW order above the thermal critical temperature is shaded yellow. Normalization of the dynamic traces according to Fig. 3b.

Supplementary Note 1: Details of the tdGL simulations of the electronic order parameter

To simulate the observed electron dynamics, we solve the following equation of motion based on the transient potential energy surface (Eq. 1)

$$\frac{\partial^2}{\partial t^2}\psi = \frac{\omega_{\mathrm{AM}}^2}{2}\Big(\big(1-\eta(t)\big)\psi - \psi^3\Big) - \gamma\frac{\partial}{\partial t}\psi\,, \tag{3}$$

which yields the order parameter $\psi(t)$ used to simulate the diffracted intensities and the in-gap photoemission intensities. The initial conditions are chosen as

$$\psi = \sqrt{1-\frac{T_{\mathrm{base}}}{T_{\mathrm{c}}}} \approx 0.84\,,$$

i.e., the static Ginzburg-Landau value corresponding to the temperature before excitation, and

$$\frac{\delta\psi}{\delta t} = 0\,.$$

We perform a global fit of the electronic in-gap dynamics $\tilde{I}_{\text{in-gap}}(t)$ over the full accessible fluence range (Fig. 2f) with the free parameters damping γ and scaling factor s of the nonthermal critical temperature, see Eq. 2, while the remaining input parameters are fixed (Supplementary Table 1). In order to fit the inverted in-gap intensity, the order-parameter simulations are normalized. To model the transient potential energy surface, see Eq. 1, we use the extracted electronic temperatures in a parametrized form, see Supplementary Note 2. We find that the maximum electronic temperature yields a good description of the initial excited potential energy shape. This is evident from the saturation of $T_{\mathrm{e,max}}$ in the high-fluence regime (see Supplementary Fig. 7c) that is accompanied by an upper limit of the initial coherent modulation frequency of the electronic order parameter. This also implies that the potential energy surface does not directly scale with the absorbed fluence $\eta \not\propto F$.

We aim to define the fit parameters as simple as possible; however, we can not reproduce the experimental data over the entire fluence- and temporal range with a single global damping constant. While γ correctly captures the initial damped modulations, a constant damping results in the reappearance

of coherent oscillations in the high-fluence regime, when the potential transforms back from the high-symmetry to the double-well shape. To prevent this, we use an alternative global fit parameter γ_{rec} during the recovery ($> 2\,\mathrm{ps}$) in the high-fluence regime ($\geq 0.3\,\mathrm{mJ\ cm^{-2}}$). In real systems, dephasing prevents the reappearance of coherent oscillations during the recovery.

We account for the inhomogeneous excitation profile, corresponding to the pump and probe spot sizes (FWHM) of $\approx 230 \times 200\,\mu\mathrm{m}^2$ and $\approx 70 \times 60\,\mu\mathrm{m}^2$, respectively, by averaging over multiple simulations with varying fluences (up to $\pm 7.5\,\%$ around the centre value). Finally, to account for the temporal resolution of the experiment, the simulations are convolved with a Gaussian (FWHM = 35 fs).

Supplementary Table 1: Parameters of the tdGL simulations

Parameter	Value	Physical meaning
$\omega_{\mathrm{AM}}/2\pi$	2.2 THz[3]	AM at 100 K
γ	4.4 THz	Damping
γ_{rec}	11.3 THz	Damping during the recovery $> 2\,\mathrm{ps}$ in the overshoot regime
T_{c}	336 K[7]	Critical temperature of the CDW
s	1.22	Scaling factor of the rescaled critical temperature T_{c}^*
$\tau_{\mathrm{ph\text{-}ph}}$	2.2 ps[8]	Decay constant of the rescaled critical temperature T_{c}^*

As illustrated in Fig. 3b, the transient CDW recovery strongly deviates from static (thermal) behaviour, which necessitates the introduction of a transiently increased critical temperature T_c^* in the tdGL simulations. To highlight the requirement of a transiently increased T_c^*, we perform additional simulations employing the constant equilibrium critical temperature T_{c}, while keeping the remaining parameters fixed as described above. As Supplementary Fig. 5 shows, this does not reproduce the experimental data, as (i) the simulated oscillation frequencies are strongly overestimated due to the increased slope of the underlying potential energy landscape (determined by $\eta(t)$) and (ii) the simulated recovery sets in only at $T_{\mathrm{e}} < T_c$ – at a significant delay with respect to the experimental data.

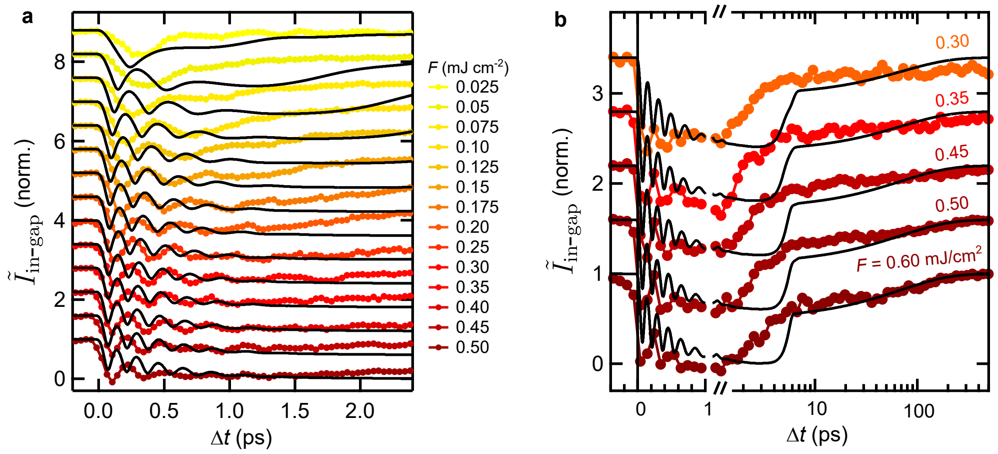

Supplementary Fig. 5: tdGL simulations using the constant equilibrium critical temperature T_{c}. (**a**) Experimental data analog to Fig. 2f and (**b**) to Fig. 3a of the main manuscript. The tdGL simulations are performed using the parameters as described above, however, using a fixed critical temperature of $T_{\mathrm{c}} = 336\,\mathrm{K}$.

Supplementary Note 2: Parametrization of the electronic temperatures

In the tdGL simulations, the electronic temperature $T_\mathrm{e}(t)$ enters as an input parameter that determines the underlying potential shape. Thus, we extract the transient electronic temperatures from Fermi-Dirac fits of the quasiparticle energy distribution of the metallic region of the FS[8,9], as these values are more reliable than the approximation by the 3TM. For each dataset, the energy resolution ($\Delta E \approx 175$ meV) is determined from a fit to energy distribution curves (EDCs) before the arrival of the pump pulse, fixing the base temperature to $T_\mathrm{base} = 100\,\mathrm{K}$. Then, the electronic temperature is extracted for varying delays, keeping the energy resolution fixed while using the position of the Fermi level and temperature as free fit parameters. Exemplary fits are shown in Supplementary Fig. 6a. A deviation from a thermal distribution appears for EDCs close to temporal pump-probe overlap, resulting in a large standard deviation of the extracted temperatures. Figure 6b depicts the electronic temperature evolution in the high-fluence regime. The relaxation of T_e features two distinct timescales, which we assign to the initial energy transfer from the electrons to specific optical phonons and a subsequent cooling of the thermalized system via diffusion.

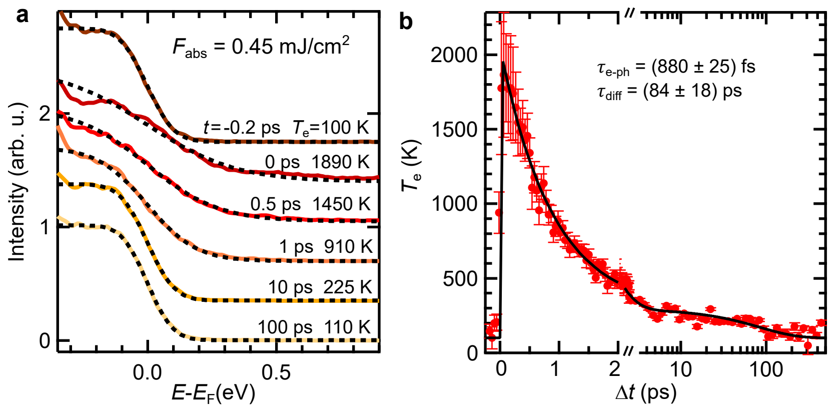

Supplementary Fig. 6: Time-dependent Fermi-Dirac fits. (**a**) EDCs extracted from the metallic region of the FS with Fermi-Dirac fits for selected pump-probe delays. (**b**) Extracted electronic temperature as function of delay with biexponential decay fit (black curve). One standard deviation of the temperature fits are given as uncertainty.

This fitting routine has been performed for all measured fluences in order to parametrize the fluence and time dependence of T_e, shown in Supplementary Fig. 7. The temporal evolution is approximated by a double-exponential decay:

$$T_\mathrm{e}(t,F) = T_\mathrm{base} + H(t)\cdot T_\mathrm{sat}(F)\left[A_0\cdot\exp(-t/\tau_\text{e-ph}) + (1-A_0)\cdot\exp(-t/\tau_\mathrm{diff})\right] \tag{4}$$

with Heaviside step function $H(t)$, the excitation-dependent temperature increase T_sat discussed below, and the amplitude ratio between the fast ($\tau_\text{e-ph}$) and slow (τ_diff) decay components. The values of the temperature parametrization are listed in Supplementary Table 2.

In the regime of strong excitation, the maximum electronic temperatures saturate at $T_\mathrm{e,max}(t \approx 0\,\mathrm{fs}) \approx 2300\,\mathrm{K}$ (see Supplementary Fig. 7c). While the electronic system has not fully thermalized close to pump-probe overlap (and therefore electronic temperatures are ill-defined), we find that this saturation trend is also evident at later pump-probe delays. This saturation effect can be either due to a highly nonlinear electronic heat capacity or due to photobleaching. As the FS of $TbTe_3$ consists of metallic and CDW-gapped regions, the electronic heat capacity is expected to follow a linear metal-like temperature dependence with an additional nonlinear increase resulting from the redistribution of spectral weight due

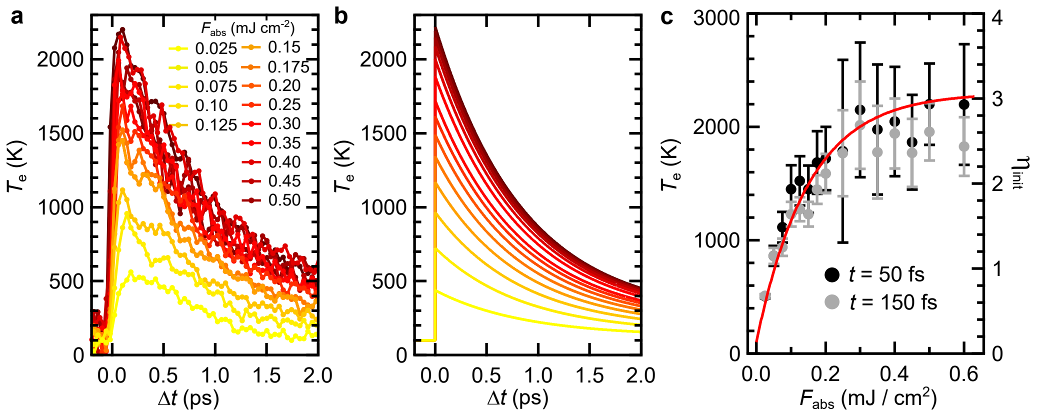

Supplementary Fig. 7: Electronic temperature parametrization. (**a**) Extracted temporal evolution of electronic temperatures and (**b**) parametrization by Supplementary Eq. 4. (c) Extracted electronic temperatures close to temporal pump-probe overlap versus fluence. The saturation model of the maximum electronic temperatures (Supplementary Eq. 6) is shown in red. One standard deviation of the temperature fits are given as uncertainty.

to the phase transition[8,10]. Furthermore, our observations agree with the saturation plateau of excited quasiparticle intensity in the related compound $LaTe_3$[11]. Such a fluence saturation trend of the electronic excitation level has also been observed in Blue Bronze[12].

We model the fluence-dependence of the temperature saturation as

$$T_{\mathrm{sat}}(F) = T_0 \cdot [1 - \exp(-F/f)]\,, \tag{5}$$

with the upper temperature limit T_0 and the fluence scaling factor f. The maximum electronic temperature is therefore given by

$$T_{\mathrm{e,max}}(F) = T_{\mathrm{base}} + T_{\mathrm{sat}}\,. \tag{6}$$

Supplementary Table 2: Parametrization values of the electronic temperature.

Parameter	Value	Physical meaning
T_{base}	100 K	Base temperature of the sample
T_0	2200 K	Temperature limit of the saturation model
f	0.15 mJ cm^{-2}	Fluence scaling factor
$\tau_{\mathrm{e\text{-}ph}}$	0.85 ps	Fast decay constant of the electronic temperature evolution
τ_{diff}	85 ps	Slow decay constant of the electronic temperature evolution
A_0	0.92	Amplitude ratio between the two components of the biexponential decay

Supplementary Note 3: tdGL simulations of the structural order parameter

To simulate the trXRD measurements of the SL peak intensity, we have to account for the contribution of sub-surface layers of varying excitation densities due to the finite pump and probe beam penetration depths. We introduce a layered model, in which the tdGL equation of motion is solved for each individual layer. The diffracted X-ray beam corresponding to the (2 10 1+q_{CDW}) SL reflection leaves the sample

at an exit angle of $\theta \approx 35°$. As the lateral CDW correlation length L_{coh}[7] is significantly larger than the effective penetration depth of the X-ray field $L_{\mathrm{coh}} \gg 2\delta_{\text{X-ray}}/\sin\theta$, interference of different layers has to be considered[13,14]. Thus, the total intensity is given by the coherent sum of all layers j of thickness d

$$I_{\mathrm{SL}}(t) \propto \left(\sum_{j=0}^{\infty} \exp\left(-jd/2\delta_{\text{X-ray}}\right) \cdot \psi_j(t) \right)^2 , \tag{7}$$

whereas the first term weights the contribution of each layer according to the X-ray penetration depth $\delta_{\text{X-ray}} = 25\,\mathrm{nm}$. The initial excitation level of the first layer $\eta_{0,\mathrm{init}}$ is calculated from the fluence-to-electronic-temperature calibration obtained from the trARPES data (see Supplementary Note 2). The attenuation of the excitation of burried layers is given by Lambert-Beer's law $\eta_{j,\mathrm{init}} = \eta_{0,\mathrm{init}} \cdot \exp(-jd/\delta_{\mathrm{pump}})$, with the penetration depth of the optical pulses $\delta_{\mathrm{pump}} = 25\,\mathrm{nm}$. We choose a layer thickness of $d = 1\,\mathrm{nm}$ and sum the 250 topmost layers. To account for the temporal resolution of the experimental setup, the simulated intensity is convolved with a Gaussian (FWHM of 160 fs). In the regime of very weak excitation, the introduced model leads to artifacts, as the rescaling of T_c^* causes an initial increase of $\psi(t)$ in cases where the electronic temperature barely increases. To avoid these simulation artifacts from buried layers at very low excitation densities, the order parameter $\psi_j(t)$ of layers j with excitation levels $\eta_{\mathrm{j,init}} < 0.25$ is fixed at the pre-excitation value $\psi_j(t < 0)$.

As we do not observe clear oscillations of I_{SL} upon strong excitation, we do not include the trXRD data in the global fitting procedure. Rather, we apply the parameters of the simulations of the electronic order parameter to the layered model. In agreement with previous studies, we find that the dominant oscillatory component of the SL peak intensity after weak excitation is a $\approx 1.7\,\mathrm{THz}$ mode[4,6]. As the AM softens upon cooling, it crosses the energy of this additional mode, leading to an anti-crossing behaviour. Due to their coupling, this phonon mode appears at the same wave vector as the CDW[3,5], see Supplementary Fig. 3. Thus, we use $\omega_{\mathrm{AM}} = 1.7\,\mathrm{THz}$ to simulate $|\psi_s|$. Further, we omit the averaging over varying fluences, used in the simulations of the electronic order parameter. The remaining parameters are adopted from Supplementary Note 1.

As discussed in the main text, surface steps may lead to unexcited sample areas. Therefore, a SL background persists even after strong excitation. To account for this, we rescale all structural intensity simulations by a global factor according to the maximum suppression of I_{SL} at the highest fluences.

This layered model captures all main experimental features of I_{SL}, see Fig. 2h. The absence of the oscillatory component in the high-fluence regime is well reproduced by the simulations, and results from the limited temporal resolution and the superposition of layers with varying excitation densities. Further, the absence of a recovery after strong excitation for several ps is in agreement with the simulations, and results from a destructive interference of the contributions of different layers with opposite sign of ψ, corresponding to opposite sides of the underlying potential[15]. In the low-fluence regime, the absolute intensities slightly deviate from the simulations. The absorbed fluence (determining the initial electronic temperature) is a highly sensitive input parameter of this model. Minor deviations between the fluence calibration of the trARPES and the trXRD setup have a major impact on the simulations. In addition, small uncertainties of the angle of incidence of the X-ray beam affect the penetration depth, a further sensitive parameter of this model.

Supplementary Note 4: Critical slowing-down of the CDW melting and recovery

Critical slowing-down is a ubiquitous signature of phase transitions close to equilibrium[16], and can also occur in a dynamical setting upon perturbation[17]. For several CDW systems, a dynamical slowing-down of the CDW melting after optical excitation in the regime of the threshold fluence has been observed[11,18]. Here, we present a further instance of a dynamical slowing-down, which we discuss within the tdGL framework.

First, we utilize the tdGL formalism to study t_{melt}, i.e., the CDW melting time, as function of excitation density, shown in Supplementary Fig. 8b. For clarity, we turn off the relaxation of the potential energy surface after excitation ($\eta = \text{const}$), fix the critical temperature T_c^*, and suppress damping. In the regime of weak perturbation, the first minimum appears at half the period of the AM. With increasing fluence, the CDW melting time increases and finally diverges at $\eta = 0.5$. In the divergent case, see Supplementary Fig. 8a, the energy gain of the excitation is just enough so that the order parameter approaches the local maximum $|\psi| \approx 0$. Close to this metastable point, the potential energy surface is fairly flat, which leads to a dynamical slowing-down. However, when using realistic simulation parameters, such as a relaxing potential energy surface, and taking into account an inhomogeneous excitation profile, the divergence is strongly reduced, and the simulated melting time agrees with the experimental data (see Supplementary Fig. 8c).

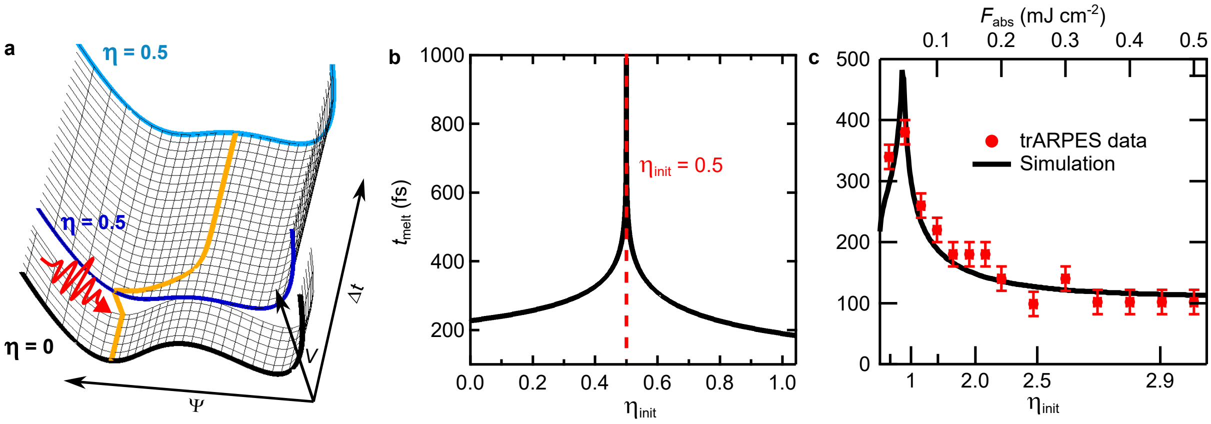

Supplementary Fig. 8: Dynamic slowing-down of the CDW melting. (**a**) Transient potential and simulated order parameter (orange line) upon excitation corresponding to $\eta = 0.5$. (**b**) Time to reach the first local minimum as function of initial excitation η_{init}. Simulation parameters of a and b: $\gamma = 0\,\text{THz}$, $T_c^* = \text{const} = 336\,\text{K}$ and $\tau_{\text{e-ph}} = \tau_{\text{diff}} = \infty$. (**c**) Initial minima of the inverted in-gap intensity, see Fig. 2f, versus absorbed fluence and initial excitation η_{init}. Results of the tdGL simulations with realistic model parameters (see Supplementary Note 1) are shown in black. The error bars of the experimentally extracted melting times represent the temporal width (FWHM) of the XUV probe pulses.

A further dynamical slowing-down can occur during the recovery of the CDW. At specific fluences, when $|\psi| \approx 0$ and $\delta\psi/\delta t \approx 0$ at the same time as the potential regains the double-well shape ($\eta = 1$), the order parameter gets frozen, illustrated in Supplementary Fig. 9. Due to the weak curvature in the vicinity of $|\psi| = 0$, the system is trapped in a metallic phase, despite an emerging double-well potential. However, this divergence is difficult to observe experimentally, as it occurs at narrow fluence windows, and is, similar to the slowing-down of the CDW melting, suppressed by crystal defects, coupling to other phonon modes and an inhomogeneous excitation profile. This critical behaviour leads to a delayed onset of CDW recovery in the simulations as compared to the electronic CDW dynamics for certain fluences, see Fig. 2f (curve $F = 0.1\,\text{mJ cm}^{-2}$) and Fig. 3a.

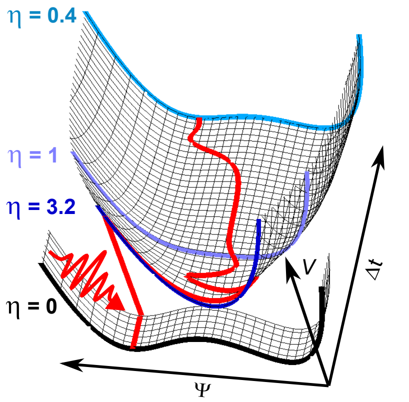

Supplementary Fig. 9: Dynamic slowing-down of the CDW recovery. Transient potential and simulated order-parameter pathway of the dynamical slowing-down during the CDW recovery. For specific excitation conditions, the order-parameter dynamics critically slow down during recovery of the CDW double-well potential. Despite the appearance of the double-well shape for $\eta < 1$ (purple line), the order parameter can get trapped at the metastable point $|\psi| \approx 0$, before it relaxes into one of the global minima. Model parameters are chosen analogous to Supplementary Note 1. To demonstrate a pronounced slowing-down, the averaging over multiple curves with varying fluences is omitted.

Supplementary References

1. Schmitt, F. *et al.* Transient electronic structure and melting of a charge density wave in $TbTe_3$. *Science* **321,** 1649–1652 (2008).
2. Schmitt, F. *et al.* Ultrafast electron dynamics in the charge density wave material $TbTe_3$. *New Journal of Physics* **13,** 063022 (2011).
3. Yusupov, R., Mertelj, T., Chu, J.-H., Fisher, I. & Mihailovic, D. Single-Particle and Collective Mode Couplings Associated with 1-and 2-Directional Electronic Ordering in Metallic RTe_3 (R= Ho, Dy, Tb). *Physical review letters* **101,** 246402 (2008).
4. Moore, R. *et al.* Ultrafast resonant soft x-ray diffraction dynamics of the charge density wave in $TbTe_3$. *Physical Review B* **93,** 024304 (2016).
5. Maschek, M. *et al.* Competing soft phonon modes at the charge-density-wave transitions in DyT e 3. *Physical Review B* **98,** 094304 (2018).
6. Trigo, M. *et al.* Coherent order parameter dynamics in $SmTe_3$. *Physical Review B* **99,** 104111 (2019).
7. Ru, N. *et al.* Effect of chemical pressure on the charge density wave transition in rare-earth tritellurides RTe_3. *Physical Review B* **77,** 035114 (2008).
8. Dolgirev, P. E. *et al.* Amplitude dynamics of the charge density wave in $LaTe_3$: Theoretical description of pump-probe experiments. *Physical Review B* **101,** 054203 (2020).
9. Wang, Y. *et al.* Measurement of intrinsic Dirac fermion cooling on the surface of the topological insulator Bi_2Se_3 using time-resolved and angle-resolved photoemission spectroscopy. *Physical Review Letters* **109,** 127401 (2012).
10. Lin, Z., Zhigilei, L. V. & Celli, V. Electron-phonon coupling and electron heat capacity of metals under conditions of strong electron-phonon nonequilibrium. *Physical Review B* **77,** 075133 (2008).
11. Zong, A. *et al.* Dynamical slowing-down in an ultrafast photoinduced phase transition. *Physical review letters* **123,** 097601 (2019).
12. Neugebauer, M. J. *et al.* Optical control of vibrational coherence triggered by an ultrafast phase transition. *Physical Review B* **99,** 220302 (2019).
13. Beaud, P. *et al.* A time-dependent order parameter for ultrafast photoinduced phase transitions. *Nature materials* **13,** 923–927 (2014).
14. Rettig, L. *et al.* Itinerant and localized magnetization dynamics in antiferromagnetic Ho. *Physical review letters* **116,** 257202 (2016).
15. Trigo, M. *et al.* Ultrafast formation of domain walls of a charge density wave in $SmTe_3$. *Physical Review B* **103,** 054109 (2021).
16. Goldenfeld, N. *Lectures on phase transitions and the renormalization group* (CRC Press, 1992).
17. Dolgirev, P. E., Michael, M. H., Zong, A., Gedik, N. & Demler, E. Self-similar dynamics of order parameter fluctuations in pump-probe experiments. *Physical Review B* **101,** 174306 (2020).
18. Tomeljak, A. *et al.* Dynamics of photoinduced charge-density-wave to metal phase transition in $K_{0.3}MoO_3$. *Physical review letters* **102,** 066404 (2009).
